# Current Practices and Variations Among Pediatric Severe Asthma Programs in the United States—The North American Severe Pediatric Asthma Consortium

**DOI:** 10.1002/ppul.71738

**Published:** 2026-07-17

**Authors:** Kristina Gaietto, Nadia Krupp, Avani V. Shah, Erhan Ararat, Samantha H. Averill, Sachin Baxi, Matejka Cernelc‐Kohan, Jeffrey M. Chambliss, Heather De Keyser, Monica Federico, Bob Geng, Akilah A. Jefferson, Parisa Kaviany, Lila C. Kertz, Kirsten Kloepfer, Allyson Larkin, Sydney Leibel, Tanya Martinez‐Fernandez, Samira Naime, Robert D. Pesek, Dinesh K. Pillai, Deepa Rastogi, Katherine Rivera‐Spoljaric, Kristie R. Ross, Franziska J. Rosser, Tregony C. Simoneau, Jade Tam‐Williams, Kelan G. Tantisira, William Anderson, Jonathan M. Gaffin, Theresa W. Guilbert, Erick Forno

**Affiliations:** ^1^ Department of Pediatrics, School of Medicine, Division of Pulmonology University of Pittsburgh Pittsburgh Pennsylvania USA; ^2^ Division of Pulmonology Allergy/Immunology and Sleep Medicine Indiana University School of Medicine Indianapolis Indiana USA; ^3^ Division Pulmonary Ann and Robert H. Lurie Chicago Northwestern University Feinberg School of Medicine Chicago Illinois USA; ^4^ Department of Pediatrics, Division of Pulmonology University of Arkansas Medical Sciences Little Rock Arkansas USA; ^5^ Department of Pediatrics Harvard Medical School, Division of Pulmonary Medicine, Boston Children's Hospital Boston Massachusetts USA; ^6^ Division of Pediatric Respiratory Medicine University of California San Diego and Rady Children's Hospital San Diego California USA; ^7^ Department of Pediatrics, Division of Pulmonology and Sleep Medicine University of Texas Southwestern Medical Center Dallas Texas USA; ^8^ Department of Pediatrics University of Colorado School of Medicine, Allergy and Immunology Section, Children's Hospital Colorado Aurora Colorado USA; ^9^ Department of Pediatrics Pediatric Pulmonary Medicine, University of Colorado School of Medicine, Children's Hospital Colorado Aurora Colorado USA; ^10^ Division of Allergy, Immunology & Rheumatology University of California San Diego and Rady Children's Hospital San Diego California USA; ^11^ Division of Pulmonary and Sleep Medicine, Children's National Hospital George Washington University School of Medicine and Health Sciences Washington District of Columbia USA; ^12^ Washington University School of Medicine, Division of Pediatric Allergy and Pulmonary Medicine St Louis Missouri USA; ^13^ Division of Allergy, Immunology, Pulmonary, and Sleep Medicine of Children's Mercy University of Missouri Kansas City Missouri USA; ^14^ Division of Respiratory and Sleep Medicine, Children's Hospital at Montefiore Albert Einstein College of Medicine New York New York USA; ^15^ Division of Pediatric Pulmonary UH Rainbow Babies and Children's/Case Western Reserve University Cleveland Ohio USA; ^16^ Department of Pediatrics, Division of Pediatric Pulmonology Cincinnati Children's Hospital Medical Center and University of Cincinnati Cincinnati Ohio USA

## Abstract

**Background:**

Severe and difficult to treat asthma in children is a complex condition causing significant morbidity and associated healthcare costs. While treatment guidelines exist for severe asthma, optimal treatment approaches for the pediatric population are less well established. Furthermore, best practices regarding real world management and implementation of guidelines for the severe pediatric asthma population are lacking.

In order to provide care for this population, institutions around the United States have developed multidisciplinary severe pediatric asthma programs (SPAPs). In recent years, the North American Severe Pediatric Asthma Consortium (NASPAC) was established as a mechanism for SPAPs to collaborate and share expertise in pediatric severe asthma. We describe the structure of the individual programs within NASPAC, as well as similarities and differences between them.

**Methods:**

Study design consisted of a cross‐sectional observational framework model, utilizing a Redcap survey that was distributed to sixteen SPAPs in 2024. Survey questions included total and new patient volume, type of staff, frequency of SPAP sessions, eligibility criteria for enrollment in the SPAP, and typical initial evaluation once enrolled. Twelve centers had completed prior surveys regarding patient volume in 2018 and 2021, and this data was used for comparison.

**Results:**

Fourteen centers responded to the 2024 survey. The centers were most similar in their incorporation of core personnel (pulmonologists, allergists, social workers, asthma educators), and the morbidity measures used for their clinic inclusion criteria. Almost all SPAPs had a basic initial evaluation including medication and technique review, adherence evaluation/discussion and spirometry. Significant variability was seen in presence of additional staff (psychologists, endocrinologists, nutritionists), and additional radiologic and laboratory testing at initial evaluation.

**Discussion:**

Significant similarities exist among fourteen independent severe pediatric asthma programs throughout the United States. However, practice variability was evident between centers. The formation of NASPAC provides opportunities for research and collaborative initiatives, as well as to establish best practices for real world management of the severe and difficult to treat pediatric asthma population.

## 1 Introduction

Asthma is one of the most common chronic diseases in children. In the United States, it affects over 5 million children and causes large economic burdens, both at the individual and population level [1, 2, 3, 4]. Severe asthma was defined over a decade ago by the American Thoracic Society (ATS)/European Respiratory Society (ERS) as asthma which requires treatment at Global Initiative for Asthma (GINA) step 4 or higher for the previous year, or systemic corticosteroids for at least half of the previous year to prevent loss of asthma control [5]. However, in the era of biologic therapies and other add‐on treatments, the definition of severe asthma can also be described as asthma that has a high treatment burden and complex treatment regimen, with less of an emphasis on oral corticosteroid need [6]. Severe asthma is estimated to affect only 2%–5% of the population [7], yet severe asthma results in high morbidity and mortality and accounts for 50% of asthma‐related healthcare costs, ensuing a high burden on children, their families, and healthcare systems [6, 8].

Severe pediatric asthma is a complex and heterogeneous disease; much about its pathophysiology remains to be understood, and effective therapies for all patients are lacking [9, 10, 11, 12, 13]. Children with severe asthma and poorly controlled disease can be categorized as severe therapy‐refractory asthma or difficult‐to‐treat (‘DTT asthma’) [6, 11]. Severe therapy‐refractory asthma is asthma in which disease pathobiology is severe and resistant to usual treatments. DTT asthma is more common than severe therapy‐refractory asthma, and is influenced by factors such as associated comorbidities, environmental exposures, medication adherence, and/or health literacy, all of which can hinder adequate asthma control [14, 15]. In practice, many children exhibit components of both severe therapy‐refractory and DTT asthma, and it is often impossible to distinguish DTT from refractory asthma at the time of initial evaluation. Therefore, we will hereafter refer to DTT and severe therapy‐refractory asthma collectively as “severe asthma.”

Many factors related to childhood asthma prevalence, control, and outcomes are highly influenced, if not contingent upon, social determinants of health (SDOH) [16]. Childhood asthma remains a health inequity, over‐burdening children living in poverty and in minoritized communities, due to inequitable distribution of both protective and harmful SDOH factors [17]. For example, persons with asthma encounter difficulties with substandard housing, poor access to high‐quality healthcare, increased exposure to air pollution, and financial constraints, all of which can worsen asthma outcomes [16, 18, 19, 20].

Given the complexity of severe asthma, a multifaceted approach to treatment through access to highly experienced multidisciplinary centers is crucial to improving outcomes. While the National Asthma Education and Prevention Program Expert Review Panel 4 (EPR‐4) 2020 updates and GINA Reports address pediatric severe asthma and discuss the importance of SDOH, management recommendations for pediatric populations are often incomplete or extrapolated from adults, and do not address best practices for implementation [15, 21, 22]. Owing to the scarcity of pediatric studies on severe asthma, at present there are no standardized, evidence‐based or consensus‐driven guidelines for severe pediatric asthma beyond proposed protocols based on current general asthma (i.e., not specifically severe asthma) or adult severe asthma guidelines [6, 23, 24, 25].

Within the last few decades, there has been a rise in the number of dedicated Severe Pediatric Asthma Programs (SPAPs) within US and international institutions. Although naming convention varies, such programs are focused on serving children with severe therapy‐refractory and DTT asthma. In 2017, asthma specialists from several SPAPs at children's hospitals across the United States founded the North American Severe Pediatric Asthma Consortium (NASPAC). The purpose of NASPAC is to share experience and collaborate in future quality improvement and research endeavors, to achieve the overall goal of improving outcomes for children with severe asthma.

In this manuscript, our objective is to describe current practice patterns at NASPAC sites in the United States. We describe similarities and differences in the multidisciplinary approach that SPAPs across the United States utilize to address pediatric severe asthma, and the scope of the population served by these clinics. We also describe annual total and new patient volumes at member SPAPs, and the range of program sizes. In addition, we describe the gaps in knowledge and resources that exist for pediatric severe asthma and the need for establishment of best practices for optimal management and implementation of guideline‐based care for this complex disease. Our hypothesis is that SPAPs will have significant commonality in core staffing, with variability in ancillary support. We also hypothesize that over time, SPAPs will demonstrate growth in yearly patient volume via addition of new patients and retention of existing patients.

## 2 Methods

NASPAC members completed a REDCap survey regarding consortium member SPAPs for calendar year 2023. One respondent was allowed per center; providers were given 4 weeks to complete the survey in July 2024. Survey questions included information about the institution (location, when SPAP was established), presence of certain types of clinic staff (e.g., social worker, respiratory therapist, psychologist), patient volume, entry criteria, referral sources, and elements included in an initial evaluation. Clinic staff included both dedicated clinic personnel and those who were available to support the SPAP but had additional responsibilities. There was some overlap in roles for some centers; for example, a clinic staff member may be in the role of program coordinator but also perform some case management duties. In this instance, centers were asked to select the one role that best described the majority of that individual's duties, so as to not count a clinic staff member more than once.

Patient volume data for 2018–2019 and 2021–2022 were also available through a prior survey and were included for comparison. We conducted descriptive bivariate analyses for all survey variables. We used linear mixed‐effects models (LMM accounting for random effects by center) to assess the differences between the distributions of total and new patients across the three survey years. All analyses were conducted in SAS (version 9.4, Cary, NC) or R Studio (version 2023.06.1 + 524).

The Indiana University School of Medicine Institutional Review Board determined that formal institutional review and informed consent were not required, as the study was not considered human subjects research. There was no funding for this study.

## 3 Results

**Figure 1:**
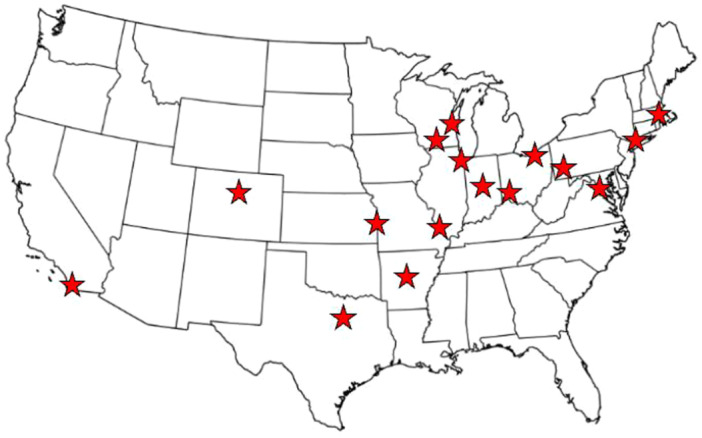
Map of NASPAC Centers. **Legend:** Location of NASPAC Centers at time of survey distribution. NASPAC centers 2024: American Family Children's Hospital*, Arkansas Children's, Boston Children's Hospital, Children's Health at UT Southwestern, Children's Hospital Colorado, The Children's Hospital at Montefiore, Children's Hospital of Wisconsin*, Children's Mercy Hospital Kansas City, Children's National Hospital, Cincinnati Children's Hospital Medical Center, Lurie Children's Hospital, Rady Children's Hospital, Riley Hospital for Children, St Louis Children's Hospital, UH Rainbow Babies and Children's Hospital, UPMC Children's Hospital of Pittsburgh. *(survey data not available). [Color figure can be viewed at wileyonlinelibrary.com]

At the time of survey distribution, NASPAC consisted of 16 centers (Figure 1). Fourteen NASPAC centers completed the 2024 survey, and their SPAPs are discussed in this report. Their SPAP characteristics are shown in Table 1. Participating centers are located throughout the United States, including East, South, Midwest and West regions, most frequently in the Midwest (43%) or East (29%). All but one SPAP was established after 2010. All SPAPs have a formal academic affiliation, have at least 50 patients, and hold severe asthma program outpatient clinic sessions at least once per month. All serve urban populations, and most (86%) also serve suburban and rural populations. All SPAPs receive internal referrals from Pediatric Pulmonology and Allergy subspecialists, and most (64%) receive referrals from other divisions within their institution (e.g. intensive care units). Fewer SPAPs receive referrals from hospital‐affiliated primary care provider practices (43%) or other practices in the city or region (29%).

**Table 1:** Severe pediatric asthma consortium (NASPAC) center characteristics—2024 survey.

Characteristics	Participating programs (*n* = 14)
Location	
East	4 (29%)
Midwest	6 (43%)
West	2 (14%)
South	2 (14%)
Year established	
2000–2010	1 (7%)
2011–2015	6 (43%)
2016–2020	5 (36%)
2021–present	1 (7%)
Clinic has formal academic affiliation	14 (100%)
Clinic is held at least once a month	14 (100%)
Clinic panel includes ≥ 50 patients	14 (100%)
Population served	
Urban	14 (100%)
Suburban	12 (86%)
Rural	12 (86%)
Has predetermined criteria for program entry	12 (86%)
Referral sources	
Internal from Pulmonology and Allergy	14 (100%)
Internal from other divisions in the hospital	9 (64%)
External from PCPs associated with the hospital	6 (43%)
External from other practices in the city/region	4 (29%)
No specific sources	1 (7%)
Number of SPAP providers per center	4 [3–6]
Pulmonologists	2 [1–4]
Allergy/Immunologists	1 [0–4]
Advance practice providers (APP)	1 [0–2]
SPAP meeting to review patients, no‐shows, QI projects, etc.	
Weekly	4 (29%)
Every other week	1 (7%)
Monthly	6 (43%)
Quarterly	2 (14%)
No regularly scheduled meeting time	1 (7%)
Number of half‐day clinic sessions/month	8 [3–24]
Physician sessions	6 [2–20]
APP sessions	2 [0–14]
Total slots/session	6 [5–15]
New slots/session	2 [1–4]
Return slots/session	4 [3–11]

*Note:* Data shown in table as number (%) or median [range].

Abbreviations: NASPAC, North American Severe pediatric asthma consortium; PCP, primary care physician; QI, quality improvement.

SPAPs have a median of four clinicians [range: 3–6], the majority of whom are pediatric pulmonologists. All centers have ≥ 1 pediatric pulmonologist; most centers also have ≥ 1 Allergist (13/14 centers) and ≥ 1 Advanced Practice Provider (APP) (10/14 centers) (Table 1). All but one SPAP has a scheduled meeting to discuss patients, review no‐shows, and work on SPAP‐related quality improvement projects; meetings occur weekly (29%), every other week (7%), monthly (43%), or quarterly (14%).

The number of clinic sessions varies significantly by center, ranging from 3 to 24 half‐day clinic sessions per month (median eight sessions/month); sessions staffed by a physician ranged from 3 to 20 per month (90%–100% of all sessions), with median of six sessions/month. Only three centers hold scheduled virtual clinic sessions (each one session per month), with the rest occurring in person. The number of patients seen per half‐day session also varies significantly by SPAP, ranging from five to 15 patients (median six patients). Most visits are for return patients who are already established at the clinic. The fourteen centers combined served a total of 5844 patients in 2023. The number of total patients seen at each SPAP in 2023 ranged from 62 to 2400 (median = 226), including 12 to 640 new patients (median = 49).

**Figure 2:**
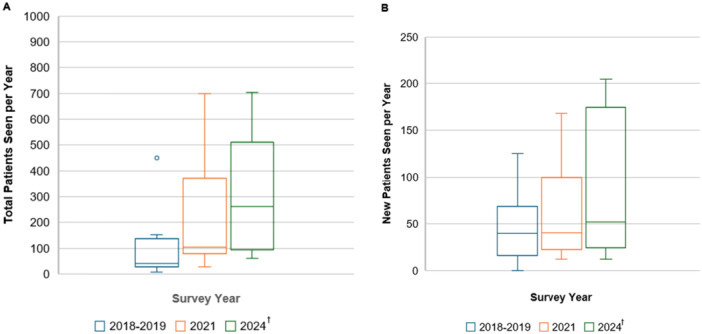
New and total patient volume 2018–2024. **Legend:** Total (panel A) and new (panel B) patient volume by survey year. Linear mixed‐effects modeling: mean increase in total patient volume = 170 patients/center/year (*p* = 0.0054); mean increase in new patient volume = 38 patients/center/year (*p* = 0.038). ^†^Outlier included in analysis but not displayed for 2024: Total patient volume = 2400, new patient volume = 640. [Color figure can be viewed at wileyonlinelibrary.com]

We then evaluated the annual patient volumes for three 12‐month periods in 2018–2019, 2021–2022, and 2023–2024. The total annual patient volume significantly increased from 2018 (median = 40, range: 9–450) to 2021 (median = 105, range: 29–700) to 2023 (median = 262, range: 62–2400); linear mixed‐effects modeling estimated the mean change was 170 additional patients per center per year (*p* = 0.0054) (Figure 2). The number of new patients similarly increased over the three periods (mean change 38 additional new patients per year, *p* = 0.038) (Figure 2). Results remained significant when the analysis was performed excluding one high‐volume outlier: mean increase was an additional 105 patients per center per year (*p* < 0.001) including an additional 19 new patients per center per year (*p* = 0.003).

The amount and type of support staff varied among centers (Table 2a). Asthma specialty nurses and social workers are included in most SPAPs (each 86%), and asthma educators, respiratory therapists, and psychologists were included in more than half. Fewer than half of SPAPs have a program coordinator, a nutritionist, a pharmacist, or a case manager. An endocrinologist, speech therapist, family health navigator, physical therapist, research assistant/QI specialist, and research coordinator were rare (each reported at only 1 SPAP).

**Table 2a:** SPAP Staff by individual center.

Component		Center														% of centers
		1	2	3	4	5	6	7	8	9	10	11	12	13	14	
Providers	Pulmonologist	X	X	X	X	X	X	X	X	X	X	X	X	X	X	100%
	Allergist	X	X	X		X	X	X	X	X	X	X	X	X	X	93%
	APP^1^		X	X	X		X	X		X	X	X	X		X	71%
Support Staff^2^	Asthma Nurse	X	X	X	X	X	X		X	X		X	X	X	X	86%
	Social Worker	X	X	X	X	X	X	X		X		X	X	X	X	86%
	Asthma Educator		X	X	X		X	X		X		X		X	X	64%
	Respiratory Therapist	X	X	X	X		X	X	X						X	57%
	Nutritionist		X					X			X		X	X		36%
	Pharmacist				X			X	X							21%
	Program Coordinator	X	X	X		X	X			X						43%
	Case Manager		X					X								14%
	Speech Therapist												X			7%
	Psychologist	X	X	X		X				X		X		X		50%

*Note:* 1‐Advanced practice provider, for example, Nurse Practitioner, Physician Assistant; 2‐does not delineate between staff to SPAP versus available

Almost all SPAPs had adopted specified entry requirements (93%), although there was variability between centers (Table 2b). A minority of centers have strict enrollment criteria, all of which needed to be met in order to qualify for the SPAP clinic (‘Required Criteria’). More commonly, centers used a combination of factors, in which patients could satisfy one or more criteria in order to be eligible for SPAP clinic (‘Qualifying Criteria’). Indicators of asthma risk and morbidity such as hospitalization and Intensive Care Unit (ICU) admissions were included by the majority of centers, while fewer than half of SPAPs had a minimum asthma controller therapy step requirement. Of the five centers that had a minimum medication requirement, three required high dose inhaled corticosteroid (ICS) with a second controller; one required medium dose ICS with a second controller and one required medium dose ICS alone. Only three centers had minimum ages (2, 4, and 5 years).

**Table 2b:** Enrollment Criteria for SPAPs by individual center.

		Center														% of centers
Component		**1**	**2**	**3**	**4**	**5**	**6**	**7**	**8**	**9**	**10**	**11**	**12**	**13**	**14**	
Entry criteria specified		X	X	X		X	X	X	X	X	X	X	X	X	X	93%
Required criteria	Minimum medication	X	X	X										X	X	36%
	Internal referral	X	X	X		X				X				X		43%
	Age		X	X		X										21%
Qualifying criteria	Hospitalization(s)	X	X	X		X	X	X	X	X	X		X	X	X	86%
	PICU^1^	X	X	X		X	X	X		X	X	X	X		X	79%
	ED visit(s)	X	X	X				X	X		X		X	X	X	64%
	Systemic corticosteroids	X		X						X	X		X	X	X	50%
	Lung function	X		X						X				X	X	36%
	School failure^2^									X						7%

*Note:* 1‐Pediatric Intensive Care Unit; 2‐excessive absence/truancy related to medical condition.

Most centers have a standard evaluation procedure, but some centers include additional work up. A basic initial SPAP evaluation at most centers includes spacer technique review, medication review, adherence review, and spirometry (each reported by 93% of centers; Table 2c). Most centers also obtain fractional exhaled nitric oxide (FeNO) and allergy testing (review or obtain) at the initial visit (each reported by 77%), as well as bronchodilator response testing and a complete blood count (CBC) with differential (each reported by 62%). Half of centers obtain a chest radiograph at the initial visit. Other components are part of a typical initial evaluation at fewer than half of the participating centers, and include behavioral health consultation, social work evaluation, nutrition consultation, plethysmography, diffusion capacity, bone density scan, and additional laboratory evaluation of immune function (Table 2c).

**Table 2c:** Standard evaluation performed at initial SPAP visit by individual center.

Component		Center														% of centers
		1	2	3	4	5	6	7	8	9	10	11	12	13	14	
Basic initial SPAP evaluation (medications, technique, adherence, spirometry)		X	X	X	X	X	X	X	X	X		X	X	X	X	93%
Pulmonary function testing	Bronchodilator response	X	X				X	X	X			X	X	X	X	64%
	Plethysmography			X												7%
	Diffusion capacity			X												7%
	FeNO^1^	X	X	X	X	X		X	X			X	X	X	X	79%
Radiology	Chest radiograph	X	X	X	X					X		X			X	50%
	Dexa scan^2^		X													7%
Laboratory	Immunoglobulins											X				7%
	Vaccine titers											X				7%
	Vitamin D											X				7%
	CBC^3^ with differential		X	X	X	X		X				X	X	X	X	64%
	ABPA^4^			X											X	14%
	Allergy testing (skin or specific IgE^5^)		X	X	X	X		X	X	X		X	X	X	X	79%
Consultations	Behavioral health		X									X		X		21%
	Nutrition		X					X					X	X		29%
	Social Work		X							X		X	X	X		36%

*Note:* 1‐Fractional exhaled nitric oxide; 2‐Dual‐Energy X‐ray Absorptiometry; 3‐Complete blood count; 4‐allergic bronchopulmonary aspergillosis evaluation (specific labs ordered may vary by institution); 5‐Immunoglobulin E.

## 4 Discussion

While severe therapy‐refractory and DTT asthma constitute a small percentage of the pediatric asthma population, such children and their families experience a disproportionately high burden of morbidity and asthma related health care expenditure in the United States [7, 8]. Addressing inequitable SDOH factors and/or challenges unique to children with severe therapy‐refractory asthma or DTT asthma often requires extra time, careful consideration, and the expertise of multiple disciplines. Recognizing the expertise and need for such care, SPAPs have arisen to provide regular follow up appointments and longitudinal assessment of outcomes that is key for this patient population [26, 27, 28].

While there are a few descriptive studies of individual SPAPs in the United States [25, 26, 29], to our knowledge, our study is the first to describe and compare multiple SPAPs simultaneously. Our survey results demonstrate that SPAPs in the United States vary in size and resources but are similar in their approach to evaluation of severe asthma. While SPAPs have been established and expanded independently, it is notable that strong similarities exist across SPAPs, particularly in terms of referral patterns, entry criteria for clinic, and evaluation. The variations between clinics have likely arisen out of a combination of necessity, with a pressing need being identified, and feasibility, in whether the institution is positioned to be able to fill that need or not. Once established, we found that these severe asthma programs have grown over time, in terms of personnel, clinic sessions, locations, and patients served, thus underscoring the ongoing need for this level of intervention.

As evidenced by our survey results, the infrastructure used to support SPAPs typically includes dedicated nursing and social work at a minimum. Almost all clinics have an established process to review individual patients, identify those who have not been seen in clinic recently or have no‐showed visits, as well as support staff including social work to facilitate transportation and address other barriers to follow up. More variable personnel include respiratory therapy, psychology, and nutrition. Multidisciplinary clinic models have previously been shown to be effective for a multitude of pediatric chronic diseases including obesity [30], congenital heart disease [31, 32], kidney disease [33], and Turner Syndrome [34]. Therefore, it is not surprising that the majority of SPAPs who participated in our survey have evolved to include at least two types of subspecialties (Pulmonology and Allergy; one also included Endocrinology) and multiple types of support staff to comprehensively serve a complex population. A limitation to our survey, however, was the lack of distinction between services available to SPAP versus services embedded within an SPAP (e.g., dedicated respiratory therapist who reviews all severe asthma patients during clinic *vs.* having a respiratory therapist available should a provider request services).

The multidisciplinary approach to severe and DTT pediatric asthma has been shown to improve certain metrics of care through prior single center studies. A study from Rady Hospital for Children found that patients who participated in their Severe Asthma Clinic for 1 year had a significant 75% reduction in emergency department (ED) visits and 73% fewer hospitalization days compared to the year prior to enrollment in the Severe Asthma Clinic [34]. Similarly, a study from UPMC Children's Hospital of Pittsburgh found that severe asthma exacerbations decreased from 3.2 to 2.2 per year after enrollment in the Severe Asthma Clinic [35]. The SPAP at Riley Hospital for Children demonstrated a 51% decrease in hospitalizations following SPAP clinic enrollment compared to the year prior, and, importantly, demonstrated both cost‐effectiveness and long‐term sustainability of the severe pediatric asthma multidisciplinary program model [35].

The concentration of high‐risk patients into the focused multidisciplinary SPAP programs has yielded important research to characterize the role of comorbidities, such as sleep disordered breathing and mental health, the prevalence of non‐steroidal anti‐inflammatory drug (NSAID) sensitivity, and the effect of non‐medical switching of controller medications on asthma control [36, 37, 38, 39, 40, 41, 42]. Nevertheless, the small populations at each individual center have precluded the ability to obtain reliable, more generalizable conclusions. NASPAC's combined patient population of over 5000 unique patients per year and wide geographic distribution throughout the US will allow for more robust research and QI initiatives for the pediatric severe and DTT asthma population. Furthermore, the NASPAC continues to add new centers, and individual centers in our study demonstrated anan increase in both total and new patient population over time, underscoring potential for growth of the NASPAC network. Since distribution of the 2024 survey, NASPAC has grown to include nineteen centers at the time of writing, with four additional affiliate centers that are in early stages of establishing SPAPs.

While the SPAPs included in this survey share many commonalities, there are significant differences in how they deliver care. The similarities seen between SPAPs suggest that standard guidelines for the management of pediatric severe asthma can be created to provide a framework for other pediatric providers who care for patients with severe asthma. By contrast, the differences between SPAPs may represent an opportunity to examine differing outcomes between centers and ultimately develop best practice guidelines for the pediatric severe therapy‐refractory/DTT asthma population. Such best practice guidelines could address SPAP structure and operations, screening practices for comorbidities such as depression, anxiety and sleep apnea, asthma treatment strategies, and transition to adult care. The availability of best practices guidelines will aid children's hospitals and pediatric asthma specialists in establishing or expanding their own severe asthma programs. While there is peer‐reviewed guidance on how to establish an adult Severe Asthma Program [43], to date, no analogous pediatric guidance has been published.

Moving forward, the size and scope of NASPAC may allow for novel investigations into severe pediatric asthma, such as the role of early life exposures, effect of social determinants of health, therapeutic effectiveness, or role of biomarkers. At present, such studies are limited by small numbers of pediatric severe asthma patients at any one institution. With expanding NASPAC membership, there is potential for the NASPAC to serve as a long term research base.

We acknowledge several limitations of our study. First, data was provided via survey and thus may be subject to recall bias. However, respondents were given 4 weeks to complete the survey and were encouraged to review granular center data so that they could accurately answer survey questions, particularly about patient volume. Second, the 14 SPAPs in our present report are each affiliated with large, free‐standing, academic children's hospitals, primarily in the Northeast and Midwest, and thus may not be representative of or generalizable to smaller, community‐based SPAPs or those in other regions of the United States. In addition, our survey results showed growth in patient volume, but our study did not explore potential factors that led to such growth. Given that most SPAPs have specific criteria for referral and the increase in the number of SPAPs over time, it is possible that improved identification of patients and program expansion were important factors, but further study aimed at evaluating the reasons for the increase in patient volume is needed to confirm this speculation. Finally, not all NASPAC member centers completed the survey, which raises the possibility of non‐response bias in our results

In sum, our descriptive report of SPAPs in the United States is the largest to date, encompassing 14 distinct centers providing care for a total of 5844 youth in 2023. The programs from the centers included in this study developed independently, but share many common features, including a multidisciplinary approach to care, enrollment criteria, referral sources, and components of the initial evaluation. Despite many commonalities, however, our study also identified significant practice variation between centers.

Future directions for NASPAC include studying centers’ outcomes and establishing best practice standards for pediatric severe asthma care, which are important ongoing needs in pediatric severe asthma care. In order to achieve this, NASPAC is currently establishing a common Redcap database for member SPAPs to input clinical and outcomes data, which incorporates standardized outcome measures to facilitate comparison of populations between centers. Next steps will be to utilize this database to examine the demographics and presenting features of SPAP patients at the time of clinic enrollment, including morbidity, lung function, and biomarkers. Future studies will utilize this database to explore patient outcomes such as hospitalization rates and emergency visit rates, as well as the real‐world effect of treatments such as biologic agents in this vulnerable population. As data collection transitions from retrospective to prospective, the multicenter framework of NASPAC will also be a platform that supports quality improvement efforts to develop best practices in not only asthma‐specific treatment strategies, but also in developing interventions in addressing health disparities in pediatric severe asthma, comorbidity evaluation and management, and mental health screening and treatment.

## Author Contributions

**Kristina Gaietto:** writing – original draft, formal analysis, writing – review and editing, investigation, software. **Nadia Krupp:** conceptualization, methodology, data curation, writing – review and editing, supervision, investigation. **Avani V. Shah:** writing – review and editing, methodology, formal analysis, investigation, software. **Erhan Ararat:** writing – review and editing, methodology, formal analysis, investigation, software. **Samantha H. Averill:** conceptualization, methodology, writing – review and editing, investigation. **Sachin Baxi:** writing – review and editing, conceptualization, investigation. **Matejka Cernelc‐Kohan:** conceptualization, writing – review and editing, investigation. **Jeffrey M. Chambliss:** conceptualization, writing – review and editing, investigation. **Heather De Keyser:** conceptualization, writing – review and editing, investigation. **Monica Federico:** conceptualization, methodology, writing – review and editing, investigation. **Bob Geng:** supervision, writing – review and editing, investigation. **Akilah A. Jefferson:** methodology, writing – review and editing, investigation. **Parisa Kaviany:** methodology, writing – review and editing, investigation. **Lila C. Kertz:** writing – review and editing, investigation. **Kirsten Kloepfer:** conceptualization, investigation, methodology, writing – review and editing. **Allyson Larkin:** conceptualization, investigation, methodology, writing – review and editing, resources. **Sydney Leibel:** investigation, writing – review and editing. **Tanya Martinez‐Fernandez:** investigation, writing – review and editing. **Samira Naime:** investigation. **Robert D. Pesek:** investigation, writing – review and editing. **Dinesh K. Pillai:** investigation, methodology, writing – review and editing. **Deepa Rastogi:** conceptualization, investigation, methodology, writing – review and editing. **Katherine Rivera‐Spoljaric:** conceptualization, investigation, writing – review and editing. **Kristie R. Ross:** conceptualization, investigation, methodology, writing – review and editing. **Franziska J. Rosser:** conceptualization, investigation, methodology, writing – review and editing. **Tregony C. Simoneau:** investigation, writing – review and editing. **Jade Tam‐Williams:** investigation, methodology, writing – review and editing. **Kelan G. Tantisira:** investigation, writing – review and editing. **William Anderson:** investigation, conceptualization, methodology, writing – review and editing. **Jonathan M. Gaffin:** conceptualization, investigation, methodology, writing – review and editing. **Theresa W. Guilbert:** conceptualization, investigation, methodology, writing – review and editing. **Erick Forno:** conceptualization, investigation, methodology, writing – review and editing, software, data curation, supervision, resources, project administration.

## Funding

The authors have nothing to report.

## Conflicts of Interest

The authors declare no conflicts of interest.

## Data Availability

The data that support the findings of this study are available from the corresponding author upon reasonable request.
